# Multiscale Engineering of Nonprecious Metal Electrocatalyst for Realizing Ultrastable Seawater Splitting in Weakly Alkaline Solution

**DOI:** 10.1002/advs.202202387

**Published:** 2022-07-07

**Authors:** Jiankun Li, Tingting Yu, Keyu Wang, Zhiheng Li, Juan He, Yixing Wang, Linfeng Lei, Linzhou Zhuang, Minghui Zhu, Cheng Lian, Zongping Shao, Zhi Xu

**Affiliations:** ^1^ State Key Laboratory of Chemical Engineering School of Chemical Engineering East China University of Science and Technology Shanghai 200237 China; ^2^ School of Chemical Engineering China University of Petroleum Qingdao 266580 China; ^3^ State Key Laboratory of Materials‐Oriented Chemical Engineering College of Chemical Engineering Nanjing Tech University Nanjing 211816 China; ^4^ WA School of Mines: Minerals Energy and Chemical Engineering (WASM‐MECE) Curtin University Perth Western Australia 6102 Australia

**Keywords:** seawater splitting, long‐term stability, hollow sphere, XAS, in situ Raman

## Abstract

Seawater electrolysis is an attractive technique for mass production of high‐purity hydrogen considering the abundance of seawater. Nevertheless, due to the complexity of seawater environment, efficient anode catalyst, that should be, cost effective, highly active for oxygen evolution reaction (OER) but negligible for Cl_2_/ClO^–^ formation, and robust toward chlorine corrosion, is urgently demanded for large‐scale application. Although catalysis typically appears at surface, while the bulk properties and morphology structure also have a significant impact on the performance, thus requiring a systematic optimization. Herein, a multiscale engineering approach toward the development of cost‐effective and robust OER electrocatalyst for operation in seawater is reported. Specifically, the engineering of hollow‐sphere structure can facilitate the removal of gas product, while atom‐level synergy between Co and Fe can promote Co sites transforming to active phase, and in situ transformation of sulfate ions layer protects catalysts from corrosion. As a result, the as‐developed hollow‐sphere structured CoFeS_x_ electrocatalyst can stably operate at a high current density of 100 mA cm^–2^ in the alkaline simulated seawater (pH = 13) for 700 h and in a neutral seawater for 20 h without attenuation. It provides a new strategy for the development of electrocatalysts with a broader application potential.

## Graphical abstract

The hollow sphere CoFeS_x_ (H‐CoFeS_x_) is developed into a highly efficient and robust seawater oxidation electrocatalyst by multiscale engineering via structural construction, in situ protection layer formation, and Fe‐doping for performance enhancement and anticorrosion. H‐CoFeS_x_ could achieve a current density of 150 mA cm^–2^ at the overpotential of 420 mV and maintained at 100 mA cm^–2^ for 700 h with no performance degradation.

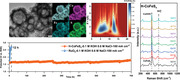

## 1 Introduction

Green hydrogen has received increasing importance in the energy source system because of its carbon‐free and high energy density (142 MJ kg^–1^).^[^ ^1^, ^2^ ^]^ Electrochemical water splitting by using renewable energy input is an attractive nonpollution technique for mass production of green hydrogen.^[^ ^3^, ^4^, ^5^ ^]^ However, commercial electrolyzers such as alkaline water electrolyzer (AWE) and proton exchange membrane water electrolyzer (PEMWE) need high‐purity water to achieve industrial hydrogen production, which makes the cost of stack and power supply account for nearly 60% of the system cost.^[^ ^6^ ^]^ Seawater or saline with low alkaline concentration could be used to replace freshwater to significantly reduce the stack and feed recycle cost, and meanwhile the dissolved salts increase the ionic conductivity of the electrolyte.^[^ ^7^, ^8^ ^]^ But there are still numerous problems that hinder the practical application of seawater splitting.^[^ ^9^ ^]^

The leading problem originates from the competing reaction of oxygen evolution reaction (OER), namely chlorine evolution reaction (ClER) in acidic environment (Equation 1) or hypochlorite evolution reaction (HCER) in alkaline (Equation 2). HCER is a two‐electron transfer reaction, with an equilibrium potential difference (versus SHE) that is ≈480 mV higher than the potential difference of ClER (130 mV). Therefore, in alkaline solution, the OER selectivity is much higher than HCER if a suitable potential (usually *V* ≤ 1.72 V versus reversible hydrogen electrode (RHE)) is applied on the cell.^[^ ^10^ ^]^

ClER:

(1) $$2{\mathrm{Cl}}^{-}\to {\mathrm{Cl}}_{2}+2e^{-}\;\;1.36V\;\mathrm{vs}.\;\mathrm{SHE},\;\mathrm{pH}=0$$

HCER:

(2) $${\mathrm{Cl}}^{-}+2{\mathrm{OH}}^{-}\to {\mathrm{ClO}}^{-}+H_{2}O\;\;0.89V\;\mathrm{vs}.\;\mathrm{SHE},\;\mathrm{pH}=14$$

Numerous non‐noble metal (such as Fe, Co, Ni, and Cu) based catalysts can be used in alkaline seawater electrolyte. Yu et al. reported a nickel‐foam (NF) supported core‐shell metal nitride catalyst with 3D structure (NiMoN@NiFeN). The amorphous layers of NiFe oxy(hydroxide) that in situ evolving from NiFeN were responsible for high selectivity and worked as protection layer for corrosion resistance to chloride. In 1.0 m KOH + 0.5 m NaCl, it achieved a current density of 500 and 1000 mA cm^–2^ at the voltages of 1.608 and 1.709 V at 60 °C.^[^ ^11^ ^]^ Song et al. synthesized a carbon‐coated sodium cobalt‐iron pyrophosphate catalyst (Na_2_Co_1‐x_Fe_x_P_2_O_7_/C, 0 ≤ *x* ≤ 1) (NCFPO/C NPs). Regulation of Co/Fe ratio further optimized the performance of OER and it needed a small overpotential of 480 mV to achieve the current density of 100 mA cm^–2^.^[^ ^12^ ^]^ Wu et al. reported a NF‐loaded heterogeneous Ni_2_P‐Fe_2_P microsheet catalyst. The bimetallic phosphide exhibited a better transfer coefficient, enhancement of electrocatalytic kinetics, and plenty of active sites with synergistic effects from Fe‐doping.^[^ ^13^ ^]^

Although attractive performance has been achieved as mentioned above, in these studies, highly concentrated alkaline electrolytes were used, which could accelerate the corrosion of electrode, bipolar plates, and catalysts, meanwhile, the large demand of alkali further increases the operation cost. Using low alkaline concentration or near neutral seawater as feed is a better option, but HCER causes severe corrosion on the catalyst and conductive substrate when the pH is lower than 13 (e.g., 0.1 m KOH + 0.5 m NaCl). Notably, most reported nonprecious metal‐based catalysts or nickel‐foam‐based self‐supported catalysts could seldom steadily operate in weakly alkaline (pH ≤ 13) or neutral NaCl‐containing environment exceeding 100 h.

To tackle the above‐mentioned challenges, rational design of catalysts with high OER selectivity, anticorrosion, and long‐term stability in weak alkaline environment is required for seawater electrolysis. Up to now, several protection strategies have proposed. For instance, Koper et al. reported that MnO_x_ electrode surface was used as a blocking layer to protect the active sites of the IrO_x_ from Cl^–^ attacking in the solution, which increased the OER selectivity but lowered the activity of the catalyst.^[^ ^14^, ^15^ ^]^ Kuang et al. presented a NF‐loaded nickel‐iron hydroxide and nickel sulfide composite (NiFe/NiS_x_‐Ni). Raman spectra and time of flight secondary ion mass spectrometry were used to verify that the sulfate and carbonate passivating layer can protect anode from being corroded by ClO^–^ during high current density operation over 1000 h.^[^ ^16^ ^]^ Badreldin et al. reported that the stronger localized Cr^*δ*+^(OOH)^*δ*−^ bonds constructed an electrostatic shielding layer to impede the anionic Cl^–^ attack.^[^ ^17^ ^]^ Considering the complexity of catalytic system, a systematic optimization of catalyst is preferred.

Herein, we report a multiscale engineering approach toward the development of cost‐effective and robust OER electrocatalyst for operation in seawater, which involves atom‐level atom synergy, surface‐level anti‐corrosion layer protection, and 3D bulk level mass transfer enhancement. We design a 3D CoFeS_x_ hollow sphere as a catalyst precursor to facilitate mass transfer,^[^ ^18^, ^19^, ^20^ ^]^ while the Co‐incorporation of Co Fe in the material realizes atom‐level synergy to optimize charge transfer and the S transformation results in the formation of a SO_4_^–^ protection layer over the catalyst surface. As a result, the derived catalyst achieves a current density of 150 mA cm^–2^ at an overpotential of 420 mV in the weakly alkaline simulated seawater (0.1 m KOH + 0.6 m NaCl) and a long‐term stability maintaining for 700 h, clearly outperforming RuO_2_ and the other reported catalysts. Moreover, after being assembled into a two‐electrode flow‐electrolyzer, H‐CoFeS_x_ could drive stable seawater electrolysis for hydrogen generation at a current density of 100 mA cm^–2^ for 100 h.

## 2 Results and Discussion

**Figure 1:**
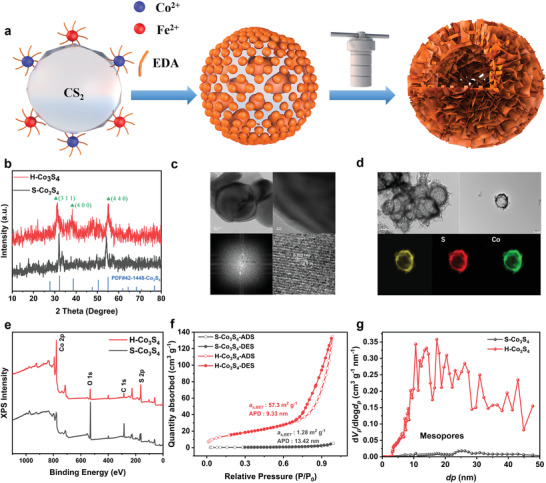
a) Synthetic illustration of the hollow sphere structure catalysts. b) X‐ray powder diffraction of H‐Co_3_S_4_ and S‐Co_3_S_4_. c) Transmission electron microscopy (TEM) images and high‐resolution TEM image of S‐Co_3_S_4_. d) TEM and mapping images of H‐Co_3_S_4_. e) X‐ray photoelectron spectroscopy (XPS) survey of S‐Co_3_S_4_ and H‐Co_3_S_4_. f) Nitrogen adsorption–desorption isotherms and g) pore size distribution of S‐Co_3_S_4_ and H‐Co_3_S_4_.

For regulating the structure of catalyst to enhance 3D bulk level mass‐transfer, the Co_3_S_4_ with solid structure (S‐Co_3_S_4_) and hollow sphere structure (H‐Co_3_S_4_) were synthesized by the reported one‐step solvothermal method.^[^ ^21^ ^]^ Carbon disulfide (CS_2_) was used as soft template and S‐source for constructing hollow structure (**Figure** **1**a). X‐ray powder diffraction (XRD) results reveal that both S‐Co_3_S_4_ and H‐Co_3_S_4_ exhibit the similar *Fd*3_*m* structure of Co_3_S_4_ (Figure 1b). The typical diffraction peaks located at 31.4°, 38.0°, and 55.0° are described to (311), (400), and (440) crystal plane of Co_3_S_4_, respectively.^[^ ^22^ ^]^ Transmission electron microscopy (TEM) further confirms the successful preparation of the solid structure of S‐Co_3_S_4_, and its cubic structure with an interplane distance of 2.03 Å for (400) facet (Figure 1c). The TEM test also verifies the successful preparation of the hollow‐sphere H‐Co_3_S_4_, whose elements homogeneously distribute throughout the sphere (Figure 1d). Meanwhile, the peaks located at 778.62 and 161.87 eV that belong to Co 2p and S 2p could be both found in the X‐ray photoelectron spectra (XPS) of H‐Co_3_S_4_ and S‐Co_3_S_4_, demonstrating their similar surface composition (Figure 1e).^[^ ^23^ ^]^ To get the detailed information about the solid and hollow sphere structure, nitrogen adsorption–desorption isotherms were used to measure their specific surface area and pore size distribution. As shown in Figure 1f, H‐Co_3_S_4_ shows a type IV adsorption–desorption isotherm with obvious hysteresis, indicating the existence of mesoporous structures.^[^ ^24^ ^]^ Meanwhile, H‐Co_3_S_4_ has a Brunauer–Emmett–Teller (BET) surface area of 57.3 m^2^ g^–1^, a pore volume of 0.21 cm^3^ g^–1^, and average pore diameter (APD) of 9.33 nm (Figure 1g, Table S3, Supporting Information), which is larger than the diameter of H_2_O (0.276 nm) and hydrated diameter of OH^–^ (0.6 nm).^[^ ^25^ ^]^ The size is large enough for the diffusing of reactant molecular into the inner space of H‐Co_3_S_4_. In contrast, the isotherm of S‐Co_3_S_4_ shows no evident hysteresis, and its BET surface area is as small as 1.28 m^2^ g^–1^, suggesting its nonporosity nature. Mesoporous channels are expected to enhance the mass‐transfer of electrolytes and reactants to the interior active sites.^[^ ^26^ ^]^

**Figure 2:**
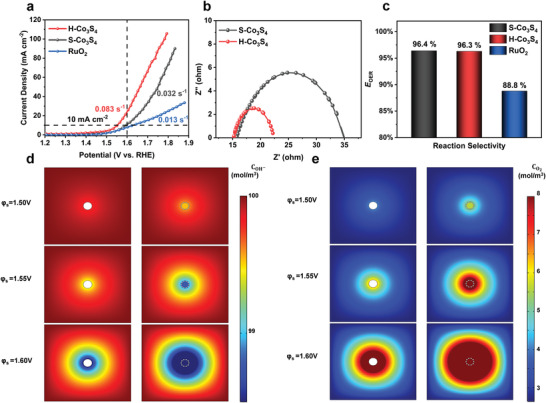
a) RDE cyclic voltammograms at mass loading of 25.51 µg cm^–2^ and a rotation rate of 1600 rpm. b) electrochemical impedance spectra (EIS) Nyquist of H‐Co_3_S_4_, S‐Co_3_S_4_, and RuO_2_. c) Reaction selectivity of H‐Co_3_S_4_, S‐Co_3_S_4_, and RuO_2_. Reactant and product concentration profiles at different potentials in two‐dimensional distribution: d) $C_{{\mathrm{OH}}^{-}}$ e) $C_{O_{2}}$.

For verifying the mass‐transfer effect from the bulk‐level modulation, seawater oxidation activities were studied by the cyclic voltammetry curves (CV) in the alkaline simulated seawater (0.1 m KOH + 0.6 m NaCl) environment. H‐Co_3_S_4_ needs a clearly lower overpotential of 322 mV than that of S‐Co_3_S_4_ (366 mV) and commercial RuO_2_ (394 mV) to achieve the current density of 10.0 mA cm^–2^ (**Figure** **2**a and Figure S1, Supporting Information). Meanwhile, the electrochemical impedance spectra (EIS) displays that the H‐Co_3_S_4_ (10.0 Ω) has a clearly lower charge transfer impedance than S‐Co_3_S_4_ (19.0 Ω) (Figure 2b). The double‐layer capacitance (*C*_dl_) tests also confirm that H‐Co_3_S_4_ has a larger electrochemically active surface area (197.5 ${\mathrm{cm}}_{\mathrm{ECSA}}^{2}$) compared with S‐Co_3_S_4_ (65.0${\;\mathrm{cm}}_{\mathrm{ECSA}}^{2}$) (Figure S2, Supporting Information). Accordingly, the turnover frequency (TOF) of H‐Co_3_S_4_ is calculated to be 0.083 s^–1^, higher than that of S‐Co_3_S_4_ (0.032 s^–1^) and RuO_2_ (0.013 s^–1^). Even S‐Co_3_S_4_ and H‐Co_3_S_4_ have nearly the same grain diameter, hollow sphere structure could enhance the performance and decrease the overpotential. The increasements of BET surface area, ECSA, and TOF confirmed that the reactants could diffuse into the hollow structure and react with the inner active sites.

Finite element method (FEM) was further used to investigate the effect of hollow sphere structure on the mass‐transfer process during electrochemical reaction. A two‐dimensional model was built up based on the experimental equipment of the electrolytic cell for simulation (Figure S4, Supporting Information). Figure 2d,e shows that on the surface of the spherical catalyst, the OH^–^ species was oxidized, producing a steady stream of O_2_ that dispersing in the diffusion domain. When the consumption rate of local OH^–^ ions exceeds the mass transport of ions in the bulk phase, the depression area of OH^–^ and accumulation area of O_2_ would appear on the electrode surface. Under the same potential of 1.6 V, obviously lower OH^–^ concentration and higher O_2_ concentration could be found near the porous hollow sphere than solid sphere (Figure S5, Supporting Information). The results indicate that the pores on the hollow sphere can promote the electrolyte to diffuse into the interior and react on the inner active sites.

Besides OER activity, the selectivity and stability are also important criteria for evaluating catalyst performance in seawater splitting. The selectivity was measured by a portable residual chlorine detector under 2.464 V versus RHE using potentiostatic method for 10 min.^[^ ^27^ ^]^ It could be known from Figure 2c that the OER selectivity of S‐Co_3_S_4_ (96.4%) and H‐Co_3_S_4_ (96.3%) are clearly higher than that of RuO_2_ (88.8%). The OER stability of the catalysts was measured under 1.664 V versus RHE with the loading of 1.0 mg cm^–2^ on the carbon paper. S‐Co_3_S_4_ and H‐Co_3_S_4_ could stably operate over 50 h, while the activity of RuO_2_ was decayed by 60% after 1.5 h (Figure S6, Supporting Information). After reaction, the hollow‐sphere structure of H‐Co_3_S_4_‐A was successfully maintained (Figure S7, Supporting Information). Amorphous region was found on the hollow sphere, which may be attributed to the surface reconstruction during the reaction.

**Figure 3:**
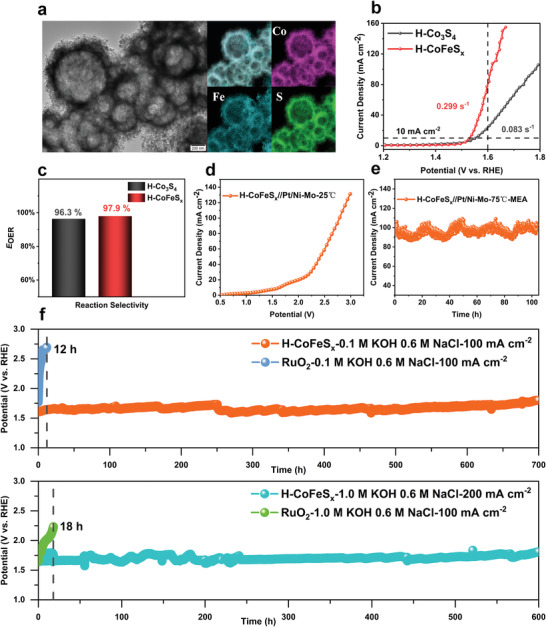
Oxygen evolution reaction (OER) performance of H‐CoFeS_x_ in alkaline seawater electrolyte. a) transmission electron microscopy (TEM) and energy ‐dispersive X‐ray spectroscopy mappings of H‐CoFeS_x_. b) RDE cyclic voltammograms at mass loading of 25.51 µg cm^–2^ and a rotation rate of 1600 rpm. c) Reaction selectivity of H‐Co_3_S_4_ and H‐CoFeS_x_. d) Cyclic voltammetry (CV) of MEA in 25 °C. e) Stability of MEA in 75 °C. f) Long‐term stability in 0.1 m KOH, 0.6 m NaCl and 1.0 m KOH, 0.6 m NaCl.

Dual‐metal active sites such as NiFe and CoFe could improve the intrinsic activity of single metal by modulating electronic structure or changing coordination environment in an atom‐level synergy interation.^[^ ^28^ ^]^ Therefore, the H‐CoFeS_x_ samples were prepared following the same procedure of H‐Co_9_S_8_, but adjusting the reactant ratio between Co(AC)_2_·4H_2_O and Fe(AC)_2_. According to the TEM and EDS‐mapping, the H‐CoFeS_x_ still possesses the hollow‐sphere structure, and the elements evenly distribute on the sphere shell (**Figure** **3**a). The mesopores could maintain after the Fe‐doping, but its BET surface area and the pore volume decrease to 44.4 m^2^ g^–1^ and 0.21 cm^3^ g^–1^, respectively (Figure S8a,b, Supporting Information), while the APD increases to 10.13 nm (Table S3, Supporting Information). FeS (PDF#15‐0037) and Co_3_S_4_ (PDF#421 448) phases are co‐existing in H‐CoFeS_x_ according to XRD pattern (Figure S9, Supporting Information). It is found that the H‐CoFeS_x_ with Co:Fe ratio of 5:5 exhibits a remarkably higher OER activity than H‐Co_9_S_8_, H‐Co_3_S_4_, and other H‐CoFeS_x_ samples with different Co:Fe ratios (Figure S10, Supporting Information).

Specifically, the current density of H‐CoFeS_x_ (CoFe55) reaches about 140 mA cm^–2^ at 1.65 V versus RHE, 3.5 times that of H‐Co_3_S_4_ (Figure 3b), and the TOF of H‐CoFeS_x_ reaches to 0.299 s^–1^ at the overpotential of 370 mV (versus RHE), higher than that of H‐Co_3_S_4_ (0.083 s^–1^). After the Fe‐doping, the charge transfer impedance of H‐CoFeS_x_ decreases from 10.0 to 4.8 Ω (Figure S11, Supporting Information), while the Tafel slope reduces from 133.0 to 60.1 mV dec^–1^ (Figure S12, Supporting Information). The ECSA of H‐CoFeS_x_ is 170.0 ${\mathrm{cm}}_{\mathrm{ECSA}}^{2}$, slightly lower than that of H‐Co_3_S_4_ (197.5 ${\mathrm{cm}}_{\mathrm{ECSA}}^{2}$), which meets well with the result of BET (Figure S13, Supporting Information). Meanwhile, its OER selectivity further increases to 97.9% (Figure 3c). The results confirm that Fe‐doping could enhance the activity, conductivity, and selectivity of catalyst. For better activity comparison with the state‐of‐the‐art catalysts in alkaline seawater OER catalysis, the performance of H‐CoFeS_x_ was also measured in 1.0 m KOH + 0.6 m NaCl. As shown in Figure S14 (Supporting Information), H‐CoFeS_x_ requires an overpotential as small as 207 mV to achieve 10 mA cm^–2^, lower than most of the reported seawater catalysts under the same circumstance (Table S4, Supporting Information). To verify the industrial application potential of H‐CoFeS_x_, a flow electrolyzer using H‐CoFeS_x_ as anode catalyst and Pt/Ni‐Mo^[^ ^29^ ^]^ as cathode catalyst was assembled (Figure S15, Supporting Information). The sweep gas was set as 83 mL min^–1^ (100 rpm) for removing the gas products from the gas diffusion electrode. The electrolyzer could operate steadily under 75 °C, keeping its current density of 100 mA cm^–2^ at the cell voltage of 2.5 V for more than 100 h in 0.1 m KOH + 0.6 m NaCl (Figure 3e). For further confirming the superior long‐term stability of the catalyst, H‐CoFeS_x_ was dropped on carbon paper to test in a three‐electrode system with 0.1 m KOH + 0.6 m NaCl and 1.0 m KOH + 0.6 m NaCl electrolytes, respectively. H‐CoFeS_x_ stably operates for 700 h at the current density of 100 mA cm^–2^ in 0.1 m KOH + 0.6 m NaCl, and 600 h at the current density of 200 mA cm^–2^ in 1. m KOH + 0.6 m NaCl. In contrast, the activity of commercial RuO_2_ decays significantly in less than 20 h under both conditions (Figure 3f). Furthermore, considering about the complex‐ion environment in the actual seawater, H‐CoFeS_x_ stability was tested in a neutral simulated seawater (0.6 m NaCl + 0.03 m SO_4_^2–^ + 0.06 m Mg^2+^ + 0.01 m Ca^2+^), whose component is closer to actual seawater referring to Strasser et al.^[^ ^8^ ^]^ As shown in Figure S16 (Supporting Information), H‐CoFeS_x_ could stably operate for about 20 h at the current density of 100 mA cm^–2^ with no obvious attenuation under such a harsh environment with high anodic oxidized potential, complex‐ion environment, and without alkali for enhancing selectivity.

**Figure 4:**
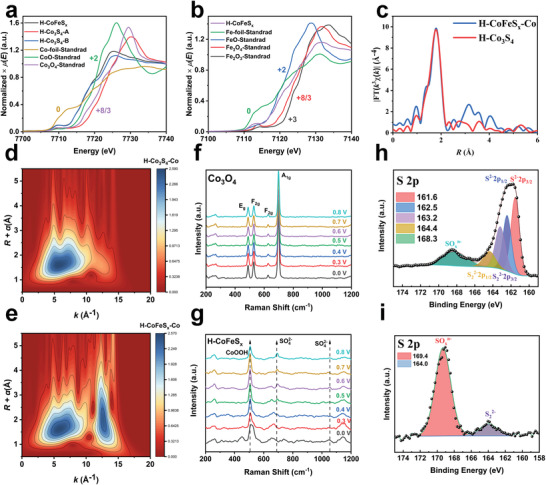
X‐ray absorption spectroscopy (XAS) of H‐Co_3_S_4_ and H‐CoFeS_x_. a) Co K‐edge X‐ray absorption near edge spectroscopy (XANES) spectra. b) Fe K‐edge XANES spectra. c) K^3^‐weighted *χ*(*k*) function of extended X‐ray absorption fine structure (EXAFS) spectra of H‐Co_3_S_4_ and H‐CoFeS_x_‐Co. d,e) Wavelet transform (WT) of the H‐Co_3_S_4_, H‐CoFeS_x_‐Co. The color in the contour indicates the moduli of the Morlet wavelet transform. f) In situ Raman spectrum of Co_3_O_4_, potential versus Ag/AgCl. g) In situ Raman spectrum of H‐CoFeS_x,_ potential versus Ag/AgCl. h,i) X‐ray photoelectron spectra (XPS) spectra of S 2p in H‐CoFeS_x_‐B and H‐CoFeS_x_‐A.

The TEM images prove that the hollow‐sphere morphology of H‐CoFeS_x_ could be maintained during electrolysis (Figure 3a, Figure S17, Supporting Information). To better understand the transformation of active sites during reaction, X‐ray absorption spectroscopy (XAS) and XPS were used to characterize the valence value of catalysts, and in situ Raman spectroscopy was used to investigate the nature of catalytic sites and the root causes of its long‐term stability and high selectivity. Several standard Co species (for example, Co foil, CoO, and Co_3_O_4_) and standard Fe species (for example, Fe foil, FeO, Fe_3_O_4_, and Fe_2_O_3_) were used as the references in the X‐ray absorption near edge spectroscopy (XANES) and extended X‐ray absorption fine structure (EXAFS) studies. According to Co XANES (**Figure** **4**a) and Fe XANES (Figure 4b), the Co K‐edge profile in H‐CoFeS_x_ is located between Co‐foil (0) and CoO (+2), nearly the same as that of original H‐Co_3_S_4_ (H‐Co_3_S_4_‐B), which is located between Co‐foil and CoO, while profile of H‐Co_3_S_4_‐A surpasses the Co_3_O_4_, demonstrating the increase of the valence state from Co*^*δ*^* ^+^ (0 < *δ* < 2) to Co*^*γ*^* ^+^ (*γ >* 2.67). The Fourier‐transformed EXAFS (FT‐EXAFS) of H‐Co_3_S_4_ Co *K*‐edge (Figure S18, Supporting Information) was carried out to examine the atomic structure and the coordination environment. A main peak at about ≈1.7 Å from the Co‐S contribution and another peak at ≈2.3 Å attributed to the Co‐S‐Co scattering path appear in the EXAFS of H‐Co_3_S_4_‐B, perfectly fitting with the Co_3_S_4_ model. While for H‐Co_3_S_4_‐A, the first peak from the contribution of Co‐O scattering path around 1.5 Å and the second peak at about 2.4 Å from the contribution of Co‐O‐Co scattering path are shown in the EXAFS, proving that Co_3_S_4_ would transform to CoOOH after the OER.^[^ ^30^ ^]^ The Fe *K*‐edge profile in H‐CoFeS_x_ is positioned between FeO (+2) and Fe_3_O_4_ (+8/3). Meanwhile, a prominent peak at 1.9 Å from the Co‐S contribution (similar to the H‐Co_3_S_4_) and another peak at about 2.6 Å from the Co‐Fe contributions appear in the Fourier transforms of H‐CoFeS_x_ (Figure 4c). It is found that the FeCo path is essential for fitting the Co *R*‐space and Fe *R*‐space data of H‐CoFeS_x_, but not for the H‐Co_3_S_4_ (Figure S19, Supporting Information). Wavelet transform (WT) analysis is an important tool for discriminating backscattering atoms, which can differentiate between heavier backscattering atoms, even they have the same distance from the central atom.^[^ ^31^ ^]^ For H‐Co_3_S_4_‐Co, only one intensity maximum is detected at about 5.8 Å^–1^ due to the Co‐S coordination (Figure 4d). With respect to the WT of H‐CoFeS_x_‐Co (Figure 4e), two intensity maximums locate at about 5.8 Å^–1^ (lighter atom) and 12.6 Å^–1^ (heavier atom). They have nearly the same coordination distance ascribing to Co‐S and Co‐Fe coordination structure, respectively, meeting well with the EXAFS of H‐CoFeS_x_.

Moreover, to verify the structure transformation process during the OER in simulated seawater, in situ Raman spectra of Co_3_O_4_ and H‐Co_3_S_4_ were applied to characterize the reaction intermediates formed on the surface of catalysts. Four different main peaks that located in 488, 530, 627, and 701 cm^–1^ match well with the E_g_, F_2g_, F_2g_, and A_1g_ vibration modes of spinel Co_3_O_4_, respectively^[^ ^32^ ^]^ (Figure 4f). No obvious change is found in the Raman spectra with the potential increasing from 0.0 to 0.8 V (versus Ag/AgCl). In contrast, in the Raman spectra of H‐Co_3_S_4_, the peaks at 457 and 525 cm^–1^ that belonging to Co‐S vibration become weaker when increasing the potential, which should be attributed to the transformation of Co_3_S_4_ to CoOOH (Figure S20, Supporting Information). The results meet well with the above‐discussed EXAFS. Moreover, during OER catalysis, the peaks around 696 and 1092 cm^–1^ that assigned to SO_4_^2–^, and the peak located at 885 cm^–1^ that attributed to S‐OH vibration show up,^[^ ^33^ ^]^ which indicates that the OH^–^ in electrolyte would combine with the S species in catalyst and transforms to SO_4_^2–^. The in situ Raman spectra of H‐CoFeS_x_ (Figure 4g) show the analogous transformation during OER in simulated seawater. The peak intensity at 510 cm^–1^ decreases with the increase of applied potential, suggesting that the CoFeS_x_ could gradually transform to CoFeOOH. A peak located at 693 cm^–1^ shows up when the applied potential reaches to 0.4 V (versus Ag/AgCl), and its intensity enhances with the further increase of applied potential, confirming the generation of SO_4_^2–^.

XPS (Figure S21a–d, Supporting Information) further showed the valence state evolution information of H‐Co_3_S_4_. In high‐resolution Co 2p spectrum (Figure S21a, Supporting Information), three fitting peaks located at 778.6, 779.8, and 784.3 eV are assigned to Co^3+^, Co^2+^ and satellite in core levels of Co 2p_3/2_.^[^ ^34^, ^35^ ^]^ After 10 h of OER stability test, the peaks in Co 2p spectrum (Figure S21b, Supporting Information) shift to 780.5 and 781.5 eV, which mainly belong to Co^3+^ cations existing in CoOOH.^[^ ^36^, ^37^ ^]^ Meanwhile, in S 2p spectrum of H‐Co_3_S_4_‐B (Figure S21c, Supporting Information), the fitted peaks that located in 161.7, 164.2, 162.8, and 163.4 eV are assigned to S^2 −^, $S_{2}^{2-}$ 2p_3/2_, S^2 −^, and $S_{2}^{2-}$ 2p_1/2_,^[^ ^38^, ^39^ ^]^ respectively, while the peak at 169.1 eV should be attributed to the slightly surface oxidation of H‐Co_3_S_4_. However, after the OER, the peak intensity in 169.3 eV experiences a significant enhancement (Figure S21d, Supporting Information), which comes from the SO_4_^2–^ passivating layer produced during OER.^[^ ^16^ ^]^ Meanwhile, the S 2p XPS (Figure 4h,i) of H‐CoFeS_x_ shows the analogous change law with H‐Co_3_S_4_, as most of S^2 −^ and $S_{2}^{2-}$ would convert into sulfate under OER oxidation potential and form a passivating layer. In the Co 2p XPS of H‐CoFeS_x_‐B (Figure S22a, Supporting Information), three different peaks that located at 778.4, 770.1, and 782.8 eV are assigned to Co^3+^, Co^2+^ and satellite in core levels of Co 2p_3/2_,^[^ ^34^, ^35^ ^]^ similar to the peaks in Co 2p XPS of H‐Co_3_S_4_‐A. While for H‐CoFeS_x_‐A (Figure S22b, Supporting Information), only two fitting peaks appear in the Co 2p XPS belonging to satellite peak and Co^3+^ in CoFeOOH.^[^ ^40^ ^]^ These results confirm that the Fe‐doping could promote the oxidation process of Co^2+^ to Co^3+^, so that nearly all Co^2+^ of H‐CoFeS_x_ could be oxidized to Co^3+^.^[^ ^41^ ^]^ Besides, two different peaks locate at 711.8 and 707.1 eV in the Fe 2p XPS of H‐CoFeS_x_‐B (Figure S22c, Supporting Information), indicating the coexistence of Fe^3+^ and Fe^2+^.^[^ ^42^ ^]^ And the intensity of Fe^3+^ peak remarkably increases after the highly oxidizing reaction (Figure S22d, Supporting Information). It has been proved that multivalent anions could improve cation selectivity and obstruct the chloride anions.^[^ ^43^ ^]^ Chen et al. reported that the addition of sulfate tends to adsorb on anode surface to form a negative charge layer in order to repulses the chloride ions away from the anode by electrostatic repulsion, further improving the corrosion resistance.^[^ ^44^ ^]^ However, the testing electrolyte needs additional 0.05 m Na_2_SO_4_ in 1.0 m KOH + seawater or 0.23 m Na_2_SO_4_ in 6,0 m KOH +2.3 m NaCl to protect the anode. We believe that higher concentration of Na_2_SO_4_ is needed in lowering the concentration of KOH electrolyte to achieve this strategy. Thus, the strategy of in situ sulfate transforming from S in H‐Co_3_S_4_ and H‐CoFeS_x_ proposed in this work may repel chloride anions and prevent them from corroding the active layer without any additives, endowing the catalysts with promising long‐term stability in seawater splitting.

## 3 Conclusion

To summarize, a multiscale engineering approach was applied in this work to endow the metal sulfide catalysts with ultrastable seawater splitting activity. Consequently, H‐CoFeS_x_ could exhibit a current density of 150 mA cm^–2^ at an overpotential of 420 mV in alkaline seawater, as well as negligible current degradation for more than 700 h at 100 mA cm^–2^ current density in the alkaline simulated seawater environment. Moreover, H‐CoFeS_x_ could stably operate for about 20 h in neutral seawater at the current density of 100 mA cm^–2^ with no obvious attenuation, demonstrating its great application potential in actual seawater. Experiments, characterizations, and simulation prove that the hollow‐sphere structure construction could improve the mass‐transfer of reactants in a 3D bulk‐level and increase the specific area of catalysts in seawater splitting. Co‐incorporation of CoFe in the material endows atom‐level synergy to optimize charge transfer and facilitates the transformation of active sites to metal (oxy)hydroxide for enhancing the activity, conductivity, selectivity, and stability of catalysts. Meanwhile, the S species in H‐Co_3_S_4_ and H‐CoFeS_x_ could in situ transform to SO_4_^2–^ during OER to provide a protecting layer for anticorrosion in a surface‐level with no extra additives. We believe that the multiscale engineering approach demonstrated in this work may be also applied for other active nonprecious metal based OER electrodes and promote the large‐scale alkaline seawater splitting to produce green hydrogen.

## 4 Experimental Section

### Materials and Instrumentation

Cobalt(II) acetate tetrahydrate (Co(AC)_2_·4H_2_O, 95%), iron(II) acetate (Fe(AC)_2_, 90%), isopropyl alcohol ((CH_3_)_2_CHOH, 98%), tricobalt tetraoxide (Co_3_O_4_, 99%), and carbon disulfide (CS_2_) were purchased from Shanghai Aladdin Biochemical Technology Co., Ltd. Sulfur flowers (S) was purchased from Alfa Aesar. Potassium hydroxide (KOH, 95%) was purchased from MACKLIN. Ethylenediamine monohydrate (C_2_H_8_N_2_·H_2_O, 98%) was purchased from Tokyo Chemical industry Co., Ltd. Sodium chloride (NaCl, 99.5%), calcium chloride (CaCl_2_, 99.5%), magnesium dichloride (MgCl_2_, 99.5%), and sodium sulfate (Na_2_SO_4_, 99.5%) were purchased from Shanghai Titan Scientific Co., Ltd. All the chemicals were used with no further purification.

### Synthesis

The S‐Co_3_S_4_ was synthesized by one‐step hydrothermal reduction method. An amount of 100 mg of S‐Co_3_O_4_, 200 mg of S powder, 150 mg of sodium borohydride powder, and 60 ml DI water were mixed, followed by magnetic stirring for 30 min, and moved into 100 mL of Teflon‐lined stainless‐steel autoclave for a reaction of 11 h at 200 °C. The S‐Co_3_S_4_ was obtained by centrifugation and alternate washing with DI water and ethanol for several times and transferred to vacuum drying chamber at 80 °C overnight.

H‐Co_3_S_4_ and H‐Co_9_S_8_ were synthesized by the method in conformity to previous study.^[^ ^21^ ^]^ H‐CoFeS_x_ and different ratio CoFe (*α*:*β*) were just changing the Co(AC)_2_·4H_2_O and Fe(AC)_2_ scale, followed by transferring to 100 mL of Teflon‐lined stainless‐steel autoclave for a reaction of 9 h at 200 °C. The product was obtained by centrifugation and alternate washing with DI water and finally lyophilized.

The HER catalyst was prepared by a two‐step method.^[^ ^29^ ^]^ Ni(NO_3_)_2_·6H_2_O, (NH_4_)_6_Mo_7_O_24_·4H_2_O, and H_2_PtCl_6_·6H_2_O were mixed in deionized water to get a 60 mL solution with 40 × 10^−3^ m of Ni, 10 × 10^−3^ m of Mo, and 0.5 × 10^−3^ m of Pt in a PTFE hydrothermal reactor. A piece of 2 × 2 cm Ni foam was placed in it, the reaction kept in 150 °C for 6 h. Second, the sample was annealed in an H_2_/Ar (v/v, 5:95) atmosphere at 500 °C for 15 min.

### Physical Characterization

XRD data was collected on a Bruker D8 Advance X‐Ray Polycrystalline Diffractometer at 40 kV/40 mA with Cu K*α* radiation (*λ* = 1.541874 Å) in the angular range of 5–80° for the 2*θ* angle. The transmission electron microscope images were taken on a HRTEM JEOL 2100F. XPS analysis was carried out on a Thermo ESCALAB 250Xi using an aluminium monochromatic X‐ray source (*hv* = 1486.6 eV, power = 150 W). Nitrogen adsorption–desorption isotherm measurements were performed using a MicrotracBEL BELSORP‐max volumetric adsorption analyzer at 77K. The specific surface area of samples was calculated via the BET method. The average pore size was calculated according to the Barrett–Joyner–Halenda method, and the pore size distribution was calculated according to the NLDFT model.

In situ Raman spectroscopy as carried out in an operando electrochemical PEEK cell (Gaoss Union, C031‐1) using a confocal Raman spectroscopy (LabRAM HR, Horiba J.Y., France) equipped with a 514.4 nm Ar^+^ laser and a high‐grade Leica microscope (long working distance objective 50×). The confocal pore size was set to 300 µm for all tests. A three‐electrode system was used, in which a Pt wire and a saturated Ag/AgCl (3.5 m KCl) electrode were served as counter and reference electrode respectively. Spectrum was calibrated prior to use based on the wavenumber of silicon 520.7 cm^–1^. Carbon paper (Suzhou Sinero Technology Co., Ltd) with 4 cm^2^ (2 × 2) was used as working electrode and catalyst was dropped on it (loading mass, 10 mg cm^–2^). The simulated seawater (0.6 m NaCl + 0.1 m KOH) was used as electrolyte for testing. During experiments, two spectrums’ data were collected under chronoamperometric mode with 75 s exposure time at 60 and 300 s, respectively.

The X‐ray absorption find structure spectra data for Co k‐edge of all samples were collected at BL14W1 station in Shanghai Synchrotron Radiation Facility (BSRF). The X‐ray absorption fine structure (XAFS) data of Fe k‐edge of all samples were collected at room temperature in fluorescence excitation mode using a Lytle detector. The acquired EXAFS data were processed according to the ATHENA module implemented in the IIRFFIT software packages.^[^ ^45^ ^]^ The EXAFS spectra were obtained by subtracting the post‐edge background from the overall absorption and then normalizing with respect to the edge‐jump step. Subsequently, *χ*(k) data for Co and Fe k‐edge were Fourier transformed to real (R) space using a hanning windows (dk = 1.0 Å^–1^) to separate the EXAFS contributions from different coordination shells. HAMA was used for the wavelet‐transform of samples.^[^ ^31^ ^]^

### Electrochemical Measurements

To accurately measure and compare the electrochemical performance of different catalysts, a rotating disk electrode configuration with a three‐electrode cell (Pine Research Instrumentation), and an Auto Lab workstation (Metrohm, Multi Autolan m204) were used to collect and analyse electrochemical data. Before dispensing on the electrode, ink was processed by ultrasonic dispersion for 1 h. An amount of 5 µL of catalyst ink was doped onto the glass carbon electrode (GCE, 5 mm of diameter) and dried in air atmosphere for 30 min to make the work electrode. Graphite rod and Ag/AgCl (3.5 m KCl) served as the counter and reference electrode, respectively. The catalyst ink consisted of 10 mg of sample, 0.1 mL of 5 wt% Nafion 117 solution, and 0.9 mL of 2‐propanol. For long‐term stability test, 100 µL of catalyst ink was doped onto the carbon paper (1 × 1.5 cm^2^, exposed area is 1 cm^2^) and dried in the air for 30 min. The electrolyte is simple simulated seawater, which contains 0.6 m NaCl and 0.1 m KOH. The neutral simulated seawater contains NaCl 0.6 m, SO_4_^2–^ 0.03 m, Mg^2+^ 0.06 m, and Ca^2+^ 0.01 m. CV cycling was performed to measure the electrochemically active surface area (ESCA) of catalysts in 0.1 m KOH to avoid without carbon black to avoid the conducting carbon black influence.^[^ ^46^ ^]^ By plotting the difference values of the current density at certain voltages against the scan rate, the linear slope that is twice of the *C*_dl_ can obtained. ESCA can be calculated according to Equation (3) by assuming a specific capacitance of 40 µF cm^–2^.

(3) $$\mathrm{ECSA}\;=\;\frac{C_{\mathrm{dl}}}{40\;\mu F\;cm^{-2}}$$

For the polarization measurements, the scan rate was 10 mV s^–1^, and the test temperature was 25 °C. All the potentials were calculated to the RHE according to the Equation (4):

(4) $$E_{\mathrm{RHE}}=\;E_{\mathrm{Ag}/\mathrm{Agcl}}+0.197+0.059\mathrm{pH}$$

The potentials were further iR‐compensated according to the following Equation (5):

(5) $$E_{\mathrm{corrected}}=E_{\mathrm{mearsured}}\;-\;iR_{s}$$

where *E*_corrected_ is the iR‐compensated potential, *E*_mearsured_ is the measured potential, *i* is the current and *R*_s_ is the equivalent series resistance measured by EIS. EIS measurements were carried out at an overpotential of 0.3 V versus Ag/AgCl reference electrode (3.5 m KCl). The frequency range of the EIS measurements is from 100 000 to 0.1 Hz at a perturbation AC voltage of 10 mV.

TOF values are calculated in OER process by using following Equation (6):

(6) $${\mathrm{TOF}}_{\mathrm{OER}}=\;\left(i\times a\right)/\left(4\times n\times F\right)$$

where *i* represents the tested current density at given overpotential, *a* is the surfaces area of the working electrode (0.196 cm^–2^), *n* is the mole number of active components on the working electrode, *F* is the Faraday constant (96 485 C mol^–1^).

### MEA Fabrication and Flow‐Electrolyzer Tests

The catalyst inks were prepared as follows. H‐CoFeS_x_ as nonprecious metal catalyst for anode, was mixed with 100 µL of 5 wt% Nafion 117 solution and 900 µL of 2‐propanol. Magnetic stirring and ultrasonication were used to get well‐dispersed ink for the catalyst suspension. Single side catalyst coated membrane (SCCM) was prepared by coating the catalyst on the membrane (Sustainion X37‐50 Grade T) with an effective area 4 cm^–2^ using hand‐spray method by a spray gun (argon as gas). The catalyst loading was 2.5 mg cm^–2^. Before the testing, the Sustainion membrane was immersed in a bath of 1 m KOH overnight to exchange the chloride ions with hydroxide ions. The SCCM was then placed between 4 cm^–2^ of carbon paper (Suzhou Sinero Technology Co., Ltd) and 4 cm^–2^ nickel foam‐based catalyst from the anode and cathode side, respectively. The MEA was then hot‐pressed under 5 bar and 140 °C for 5 min. MEA was placed between the home‐made bipolar plates for the flow‐electrolyzer tests.

### Methods for Detecting OCl^–^

A portable residual chlorine detector (YL‐2AZ, Shanghai Haiheng Electromechanical Instrument Co., Ltd.) was used to detect the content of ClO^–^ in aqueous solution. An amount of 500 µL of indicator was immediately added into 10 mL of sample solution, and another 1050 µL of 0.5 m H_2_SO_4_ was added, and gently shaken for 10 s. Then, the concentration of ClO^–^ will be obtained directly by putting it into the equipment. According to standard colorimetric method (as following equation), the indicator of colorless 3,3’‐dimethylbenzidine will be oxidized to yellow benzoquinone compounds when ClO^–^ is present in solution (pH < 4).^[^ ^27^ ^]^ The characteristic absorption peak of yellow compounds at about 437 nm will be caught by this detector which has already setup with the standard curve and computation program.


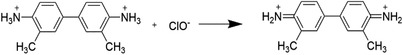


The above sample solution was obtained after 10 min chronoamperometric determination at 0.7 and 1.5 V versus Ag/AgCl. The OER Faradaic efficiencies can be calculated in terms of following Equation (7).^[^ ^10^, ^29^ ^]^ In this system, it is assumed that the total current transition efficiency is 100% and neglect the current consumption by equipment and reaction system.

(7) $$E_{\mathrm{OER}}\;\left(\%\right)=\left(1-\frac{F\times n_{{\mathrm{ClO}}^{-}}\times Z_{{\mathrm{ClO}}^{-}}}{Q}\right)\;\times 100\%$$

where *F* is Faraday constant (96 485.3 C mol^–1^), $n_{{\mathrm{ClO}}^{-}}$ and $Z_{{\mathrm{ClO}}^{-}}$ is the moles and electron transition number (2) of ClO^–^, respectively, *Q* is the total charge passed during the stability test and obtained from the electrochemical measurements.

## Conflict of Interest

The authors declare no conflict of interest.

## Supporting information

Supporting Information

## Data Availability

Research data are not shared.
