# Supplementary material for: Multiscale Engineering of Nonprecious Metal Electrocatalyst for Realizing Ultrastable Seawater Splitting in Weakly Alkaline Solution
Source: Adv Sci (Weinh). 2022 Jul 7;9(25):2202387. doi: 10.1002/advs.202202387 (PMC9443442; doi:10.1002/advs.202202387)
Supplement: Supplementary file 1 — Supporting Information [file ADVS-9-2202387-s001.pdf]

ADVANCED SCIENCE
Open Access

Supporting Information

for *Adv. Sci.*, DOI 10.1002/advs.202202387

Multiscale Engineering of Nonprecious Metal Electrocatalyst for Realizing Ultrastable Seawater Splitting in Weakly Alkaline Solution

Jiankun Li, Tingting Yu, Keyu Wang, Zhiheng Li, Juan He, Yixing Wang, Linfeng Lei, Linzhou Zhuang, Minghui Zhu, Cheng Lian, Zongping Shao* and Zhi Xu**

Supporting Information

Multi-scale Engineering of Nonprecious Metal Electrocatalyst for Realizing Ultra-stable Seawater Splitting in Weakly Alkaline Solution

Jiankun Li, Tingting Yu, Keyu Wang, Zhiheng Li, Juan He, Yixing Wang, Linfeng Lei, Linzhou Zhuang, Minghui Zhu, Cheng Lian, Zongping Shao*, and Zhi Xu**

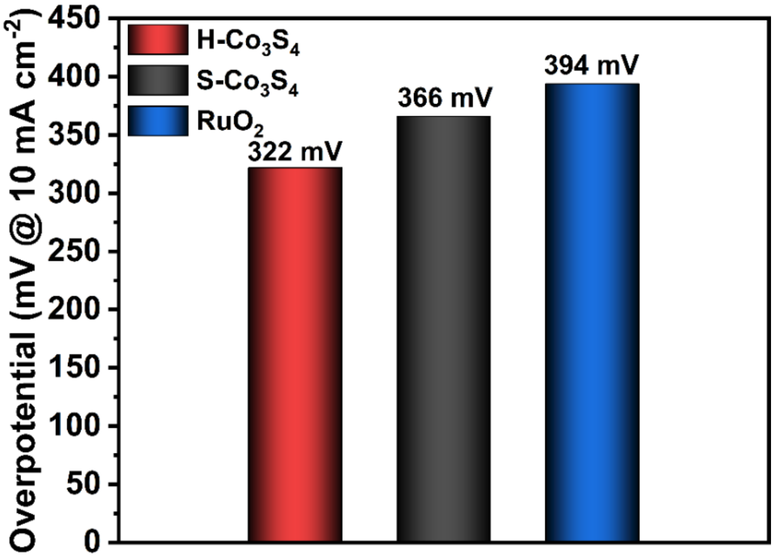

Figure S1. Overpotential of catalysts at 10 mA cm^{-2}.

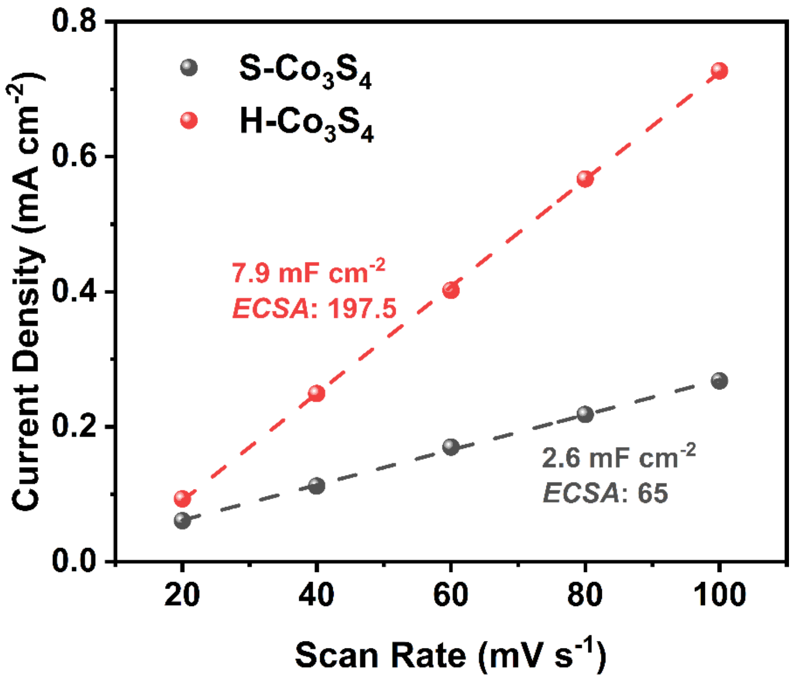

Figure S2. Double layer capacitance (Cdl) and electrochemical active surface area (ECSA) of S-Co_3S_4, H-Co_3S_4 in the non-faradaic region in 0.1 M KOH.

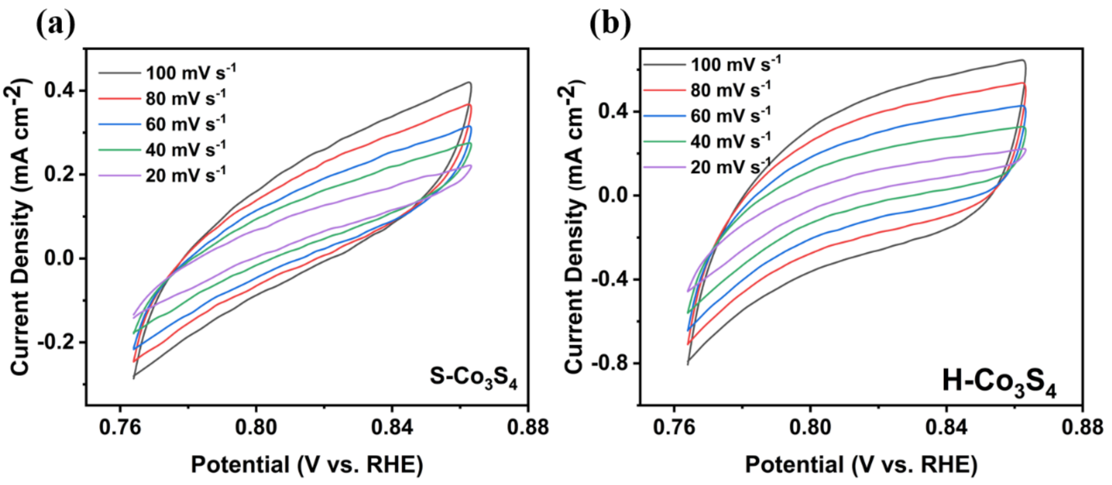

Figure S3. CV curves at different scan rates (20, 40, 60, 80, 100 mV/s) of a) S-Co_3S_4. b) H-Co_3S_4.

To explore whether a hollow sphere structure could promote the mass transfer on the catalyst or not, a diffusion model (Fick's second law, as shown as equation (1)) was used to simulate the ionic concentration using COMSOL Multiphysics software.

$$\frac{\partial}{\partial x}\left(-D_i\frac{\partial c_i}{\partial x}\right) = -\frac{\partial c_i}{\partial t} \tag{1}$$

where C_i is the concentration of species i, D_i is the diffusion coefficient of species i.
The OER in alkaline environment (equation (2)) is considered to be occurring at the planar anode surface in the reaction process.

$$4OH^- \rightarrow O_2 + 2H_2O + 4e^- \tag{2}$$

Applying overpotential is necessary for OER, and the reaction rate of such electrochemical process is a strong function of the applied potential. Generally, the OER process can be described by Butler-Volmer equation[1] (equation (3)):

$$j = j_0\left[\frac{C_R^S}{C_R^*}exp\left(\frac{\alpha_a F\eta}{RT}\right) - \frac{C_O^S}{C_O^*}exp\left(\frac{-\alpha_c F\eta}{RT}\right)\right] \tag{3}$$

where j is the current density, j_0 is the exchange current density, c^S is the species concentration near the cathode/anode, c^* is the species concentration in the bulk, α is the cathodic/anodic charge transfer coefficient, generally in this equation, $\alpha_c + \alpha_a = 1$[2]. The bulk concentrations were measured experimentally and implemented in the model. The simulation parameters can be found in Table S1.
We abstract the two-dimensional model from the experimental equipment of electrolytic cell, as shown in Figure S4. In the FEM, we respectively put the solid/hollow sphere catalyst in a 5 μm×5 μm diffusion domain. It is assumed that the ion concentration is consistent with the bulk concentration at the edge of the area. We have plotted this geometric model in COMSOL Multiphysics software.

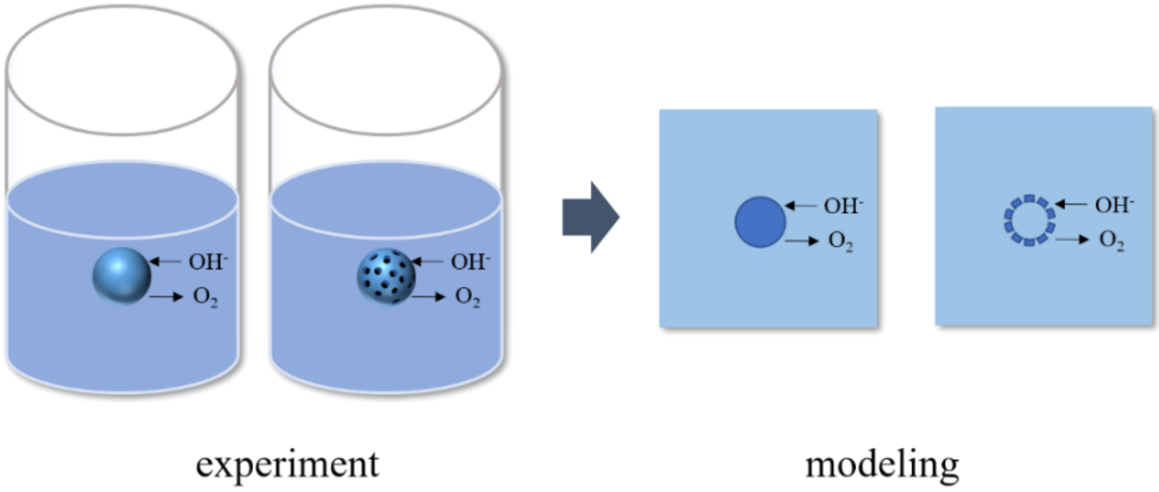

Figure S4. The establishment of two-dimensional geometric model.

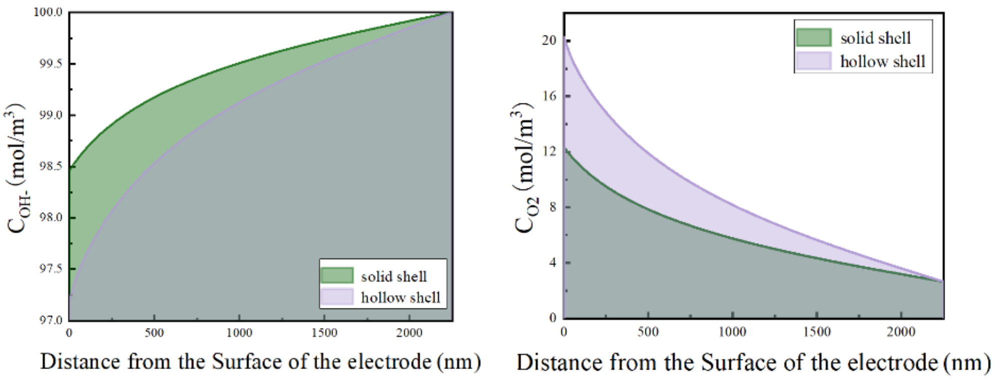

Figure S5. Ion concentration distribution at different distances from electrode surface a) C_{OH^-}, b) C_{O^2} (φs=1.6V)

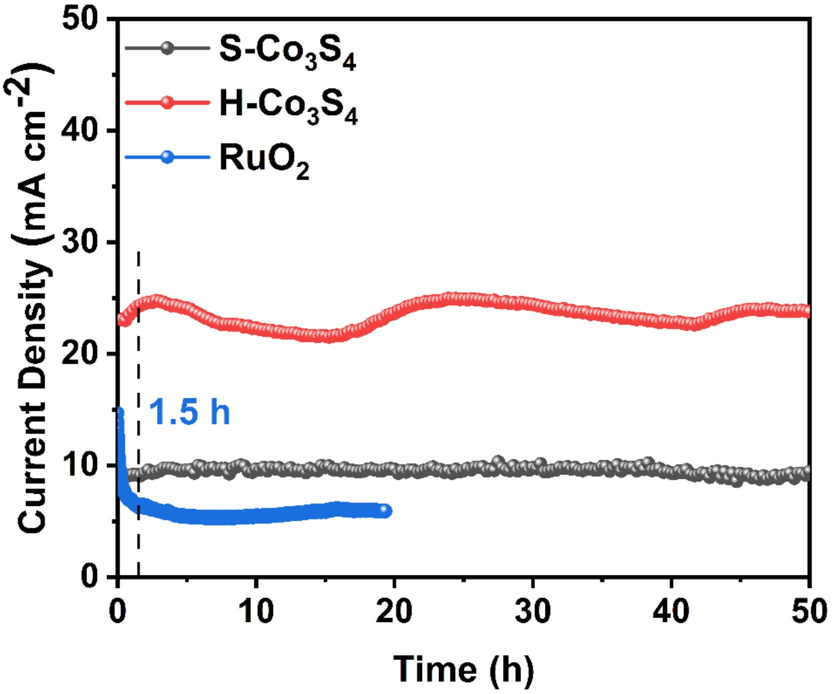

Figure S6. OER stability of the catalysts under 1.664 V vs. RHE with loading of 1.0 mg cm^{-2} on the carbon paper.

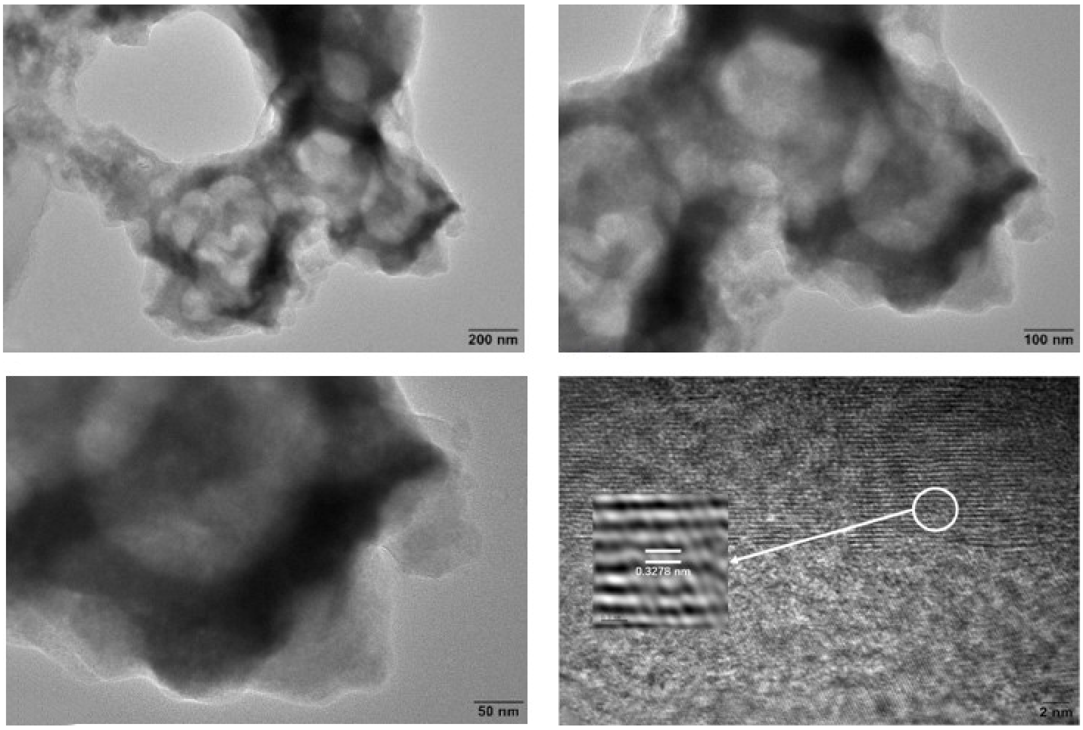

Figure S7. TEM and HRTEM of H-Co_3S_4-A.

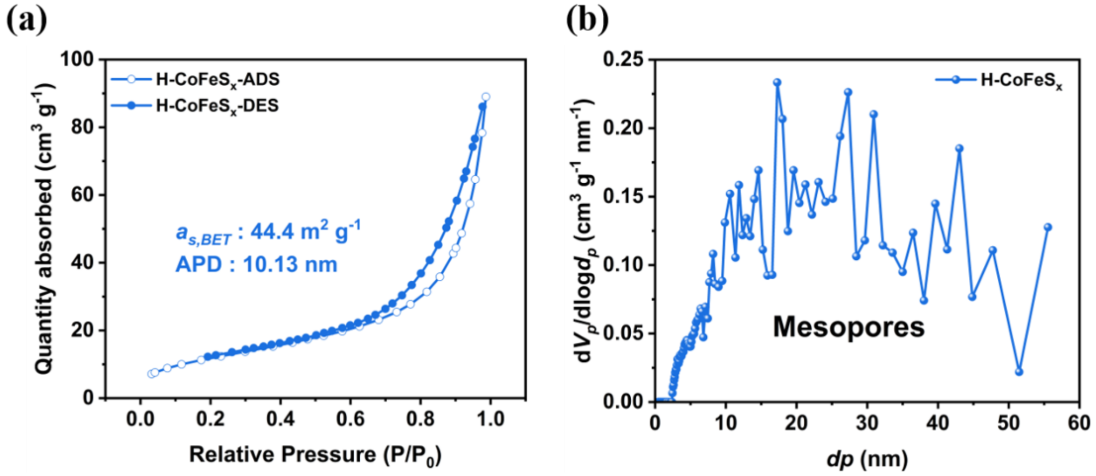

Figure S8. a) Nitrogen adsorption-desorption isotherms and b) pore size distribution of the H-$CoFeS_x$.

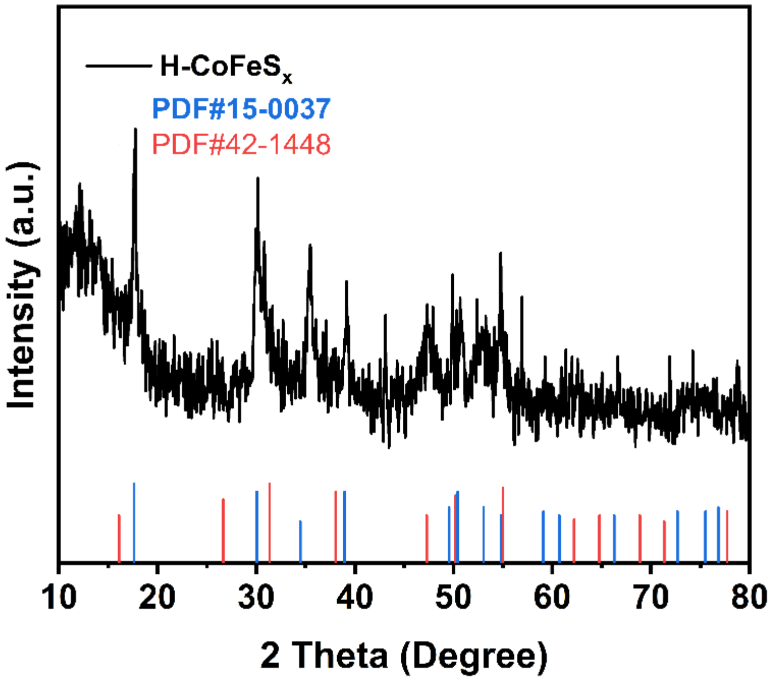

Figure S9. XRD of H-CoFeS$_x$.

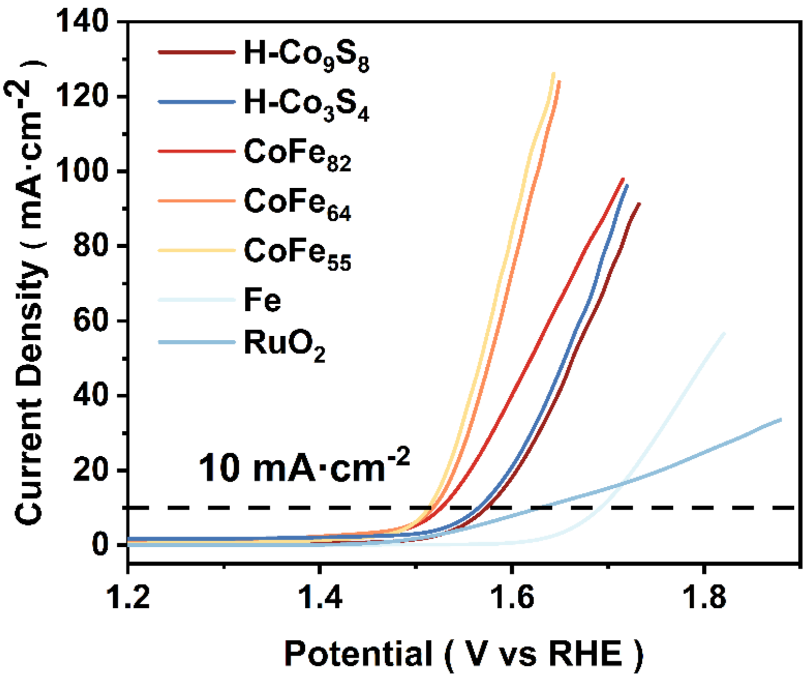

Figure S10. Regulation of Co:Fe ratio and RDE cyclic voltammograms.

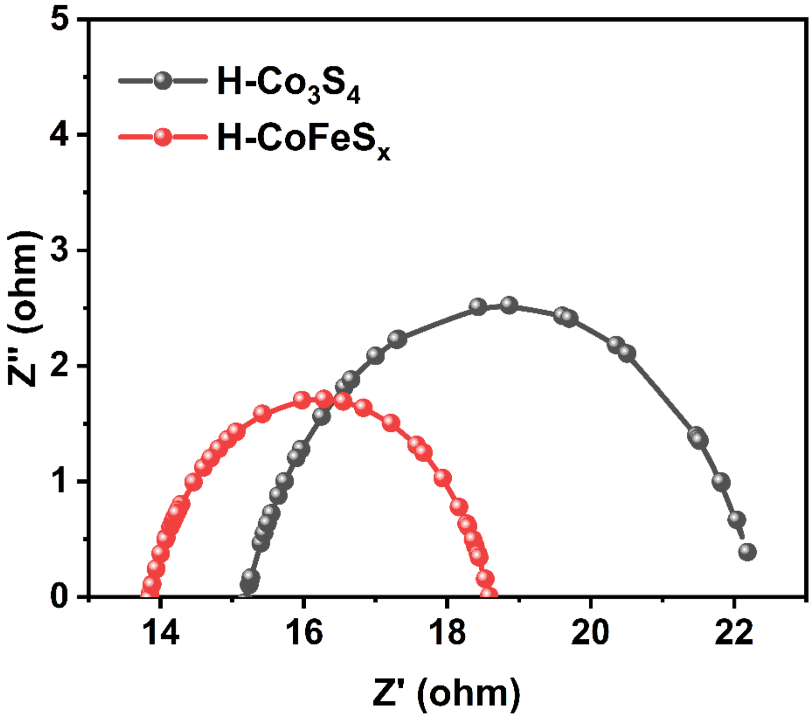

Figure S11. EIS Nyquist of H-Co_3S_4 and H-$CoFeS_x$.

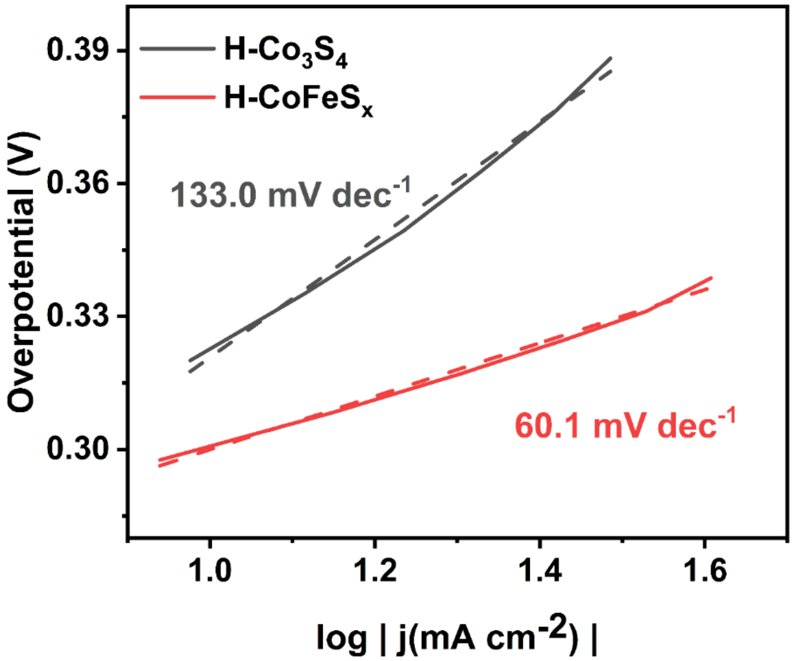

Figure S12. Tafel slope of H-Co_3S_4 and H-$CoFeS_x$.

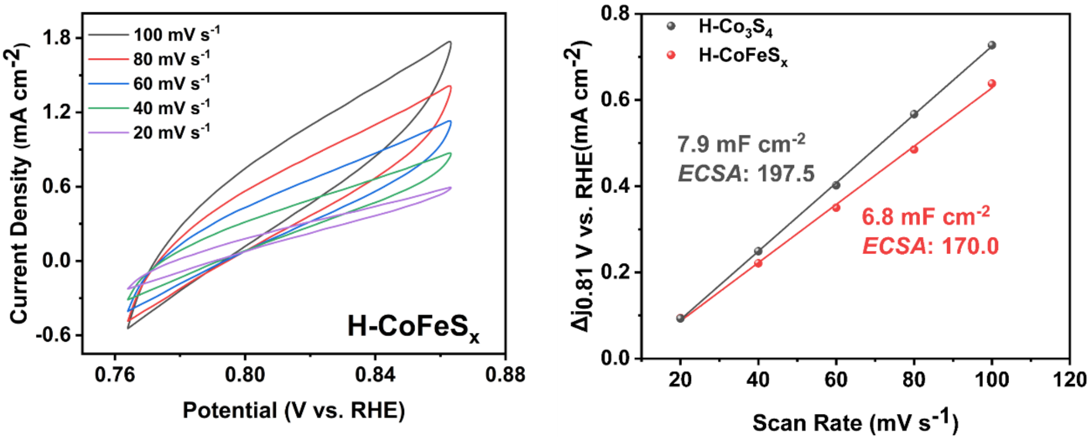

Figure S13. CV curves at different scan rates (20, 40, 60, 80, 100 mV/s), Cdl, and ECSA of H-$CoFeS_x$.

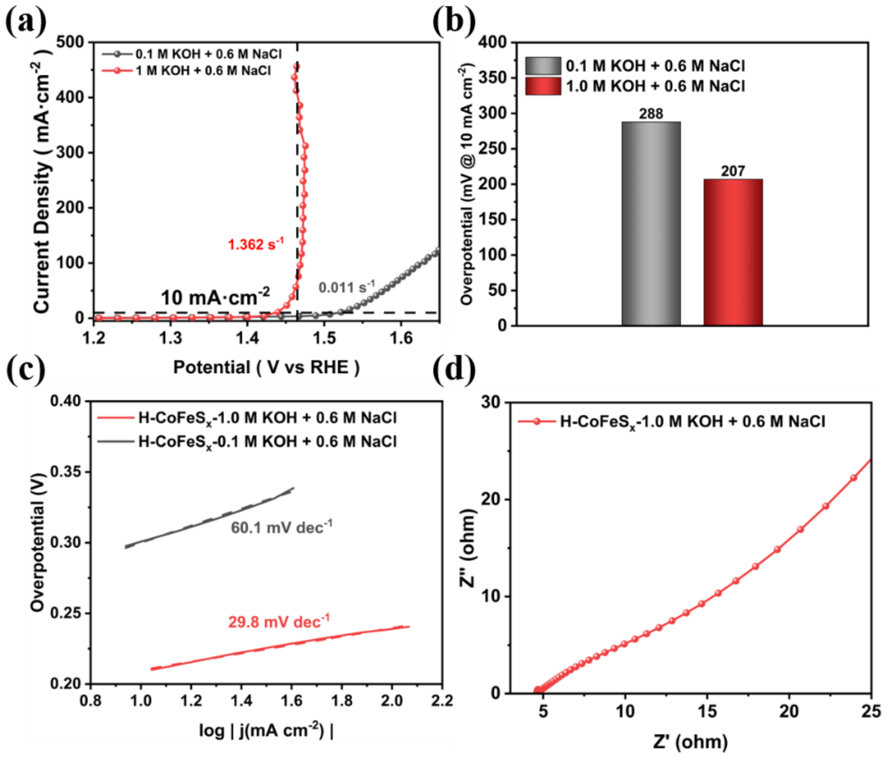

Figure S14. a) CV and TOF of H-$CoFeS_x$ in 0.1 M KOH + 0.6 M NaCl and 1.0 M KOH + 0.6 M NaCl. b) overpotential of H-$CoFeS_x$ in 0.1 M KOH + 0.6 M NaCl and 1.0 M KOH + 0.6 M NaCl at the current density of 10 mA cm^{-2}. c) Tafel slope of H-$CoFeS_x$ in 0.1 M KOH + 0.6 M NaCl and 1.0 M KOH + 0.6 M NaCl. d) EIS of H-$CoFeS_x$ in 1.0 M KOH + 0.6 M NaCl at 0 V (vs. Ag/AgCl).

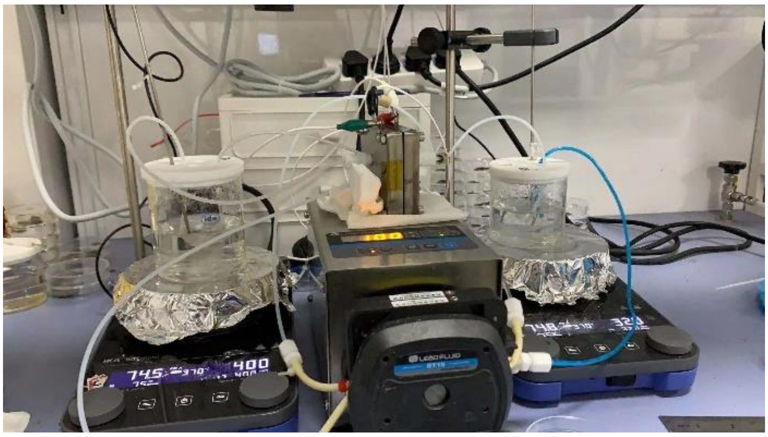

Figure S15. The photo of the flow-cell device.

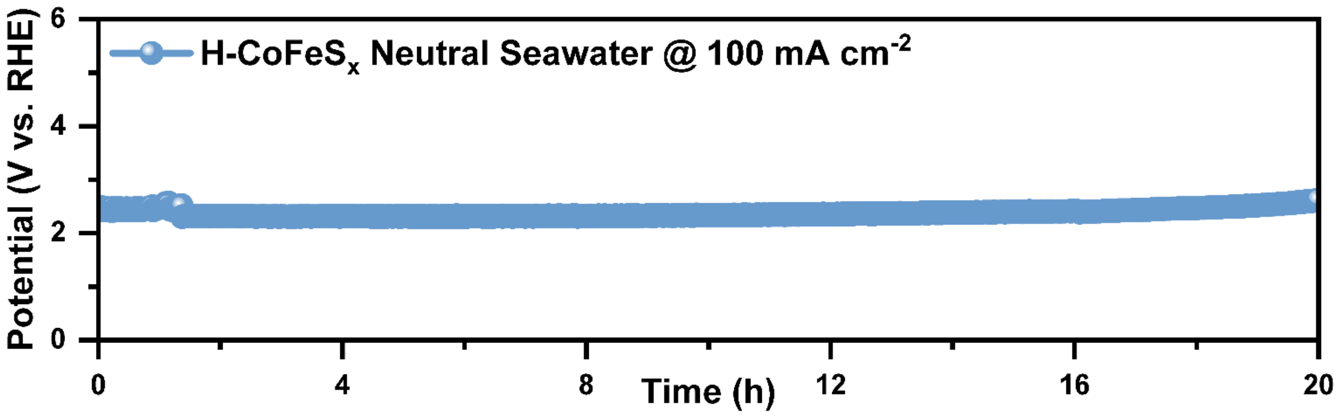

Figure S16. Stability of H-$CoFeS_x$ in a neutral simulated seawater at the current density of 100 mA cm^{-2}.

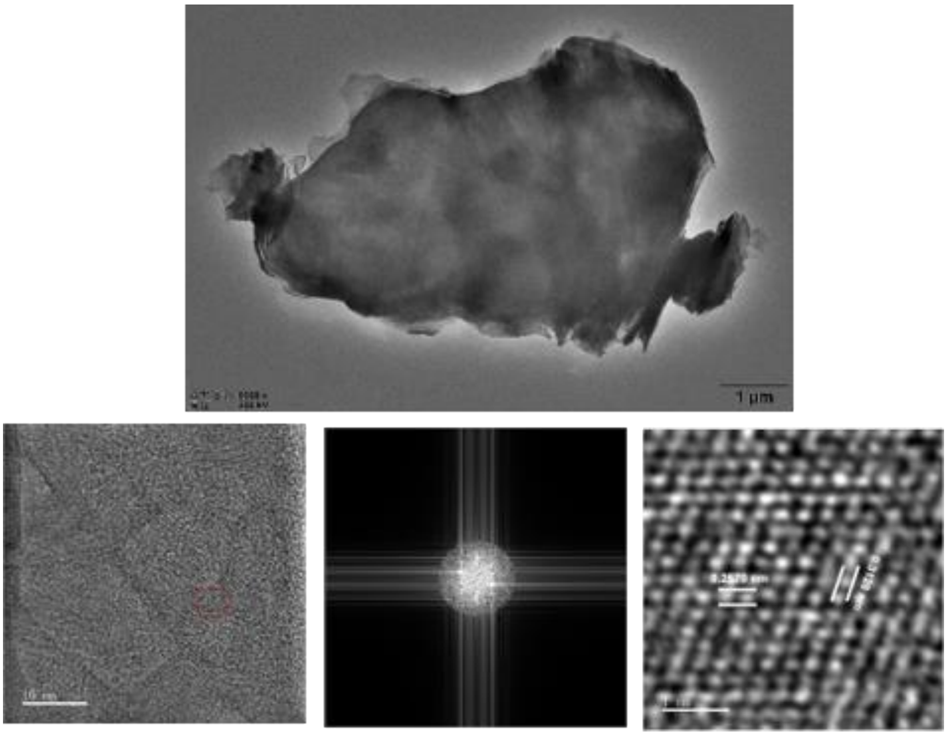

Figure S17. TEM and HRTEM of H-$CoFeS_x$-A.

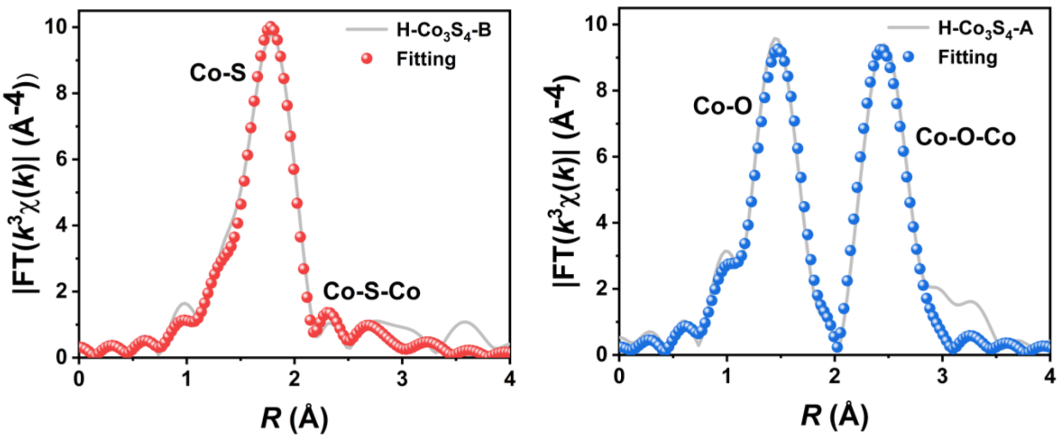

Figure S18. K^3-weighted χ(*k*) function of EXAFS spectra of H-Co_3S_4-B and H-Co_3S_4-A.

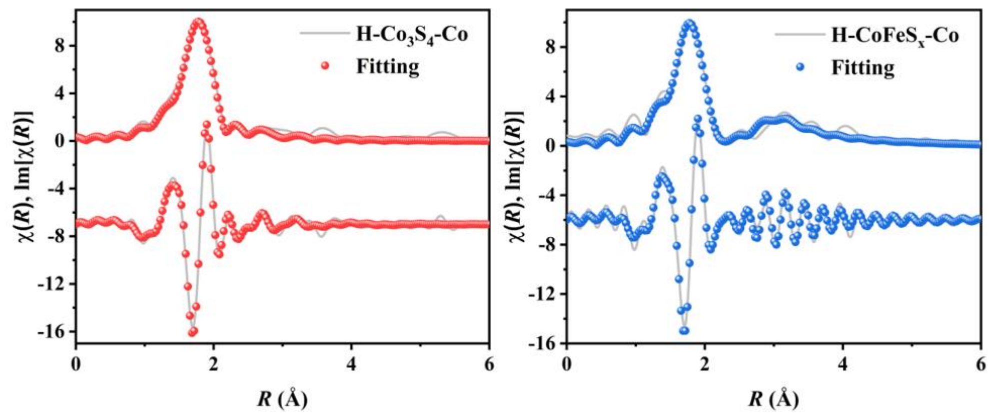

Figure S19. The corresponding Co K-edge EXAFS fitting curves of H-Co_3S_4 and H-$CoFeS_x$-Co.

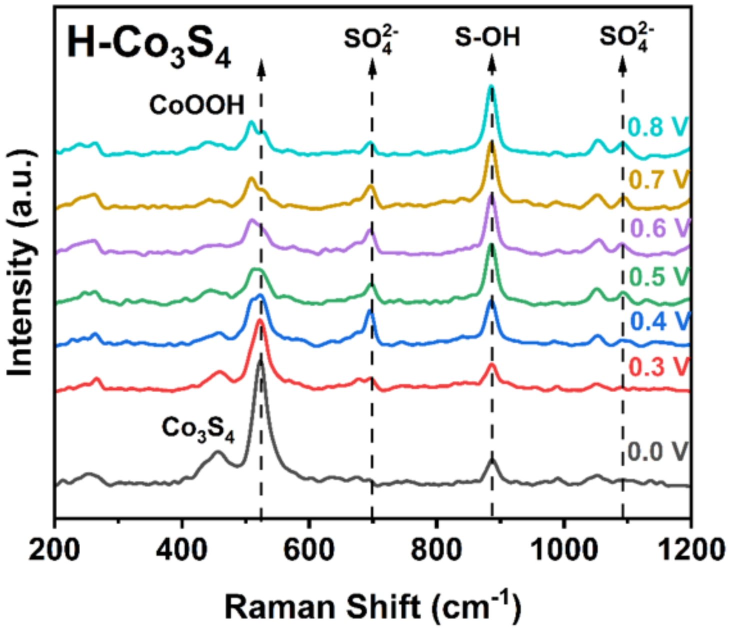

Figure S20. In-situ Raman spectrum of H-Co_3S_4, potential vs. Ag/AgCl.

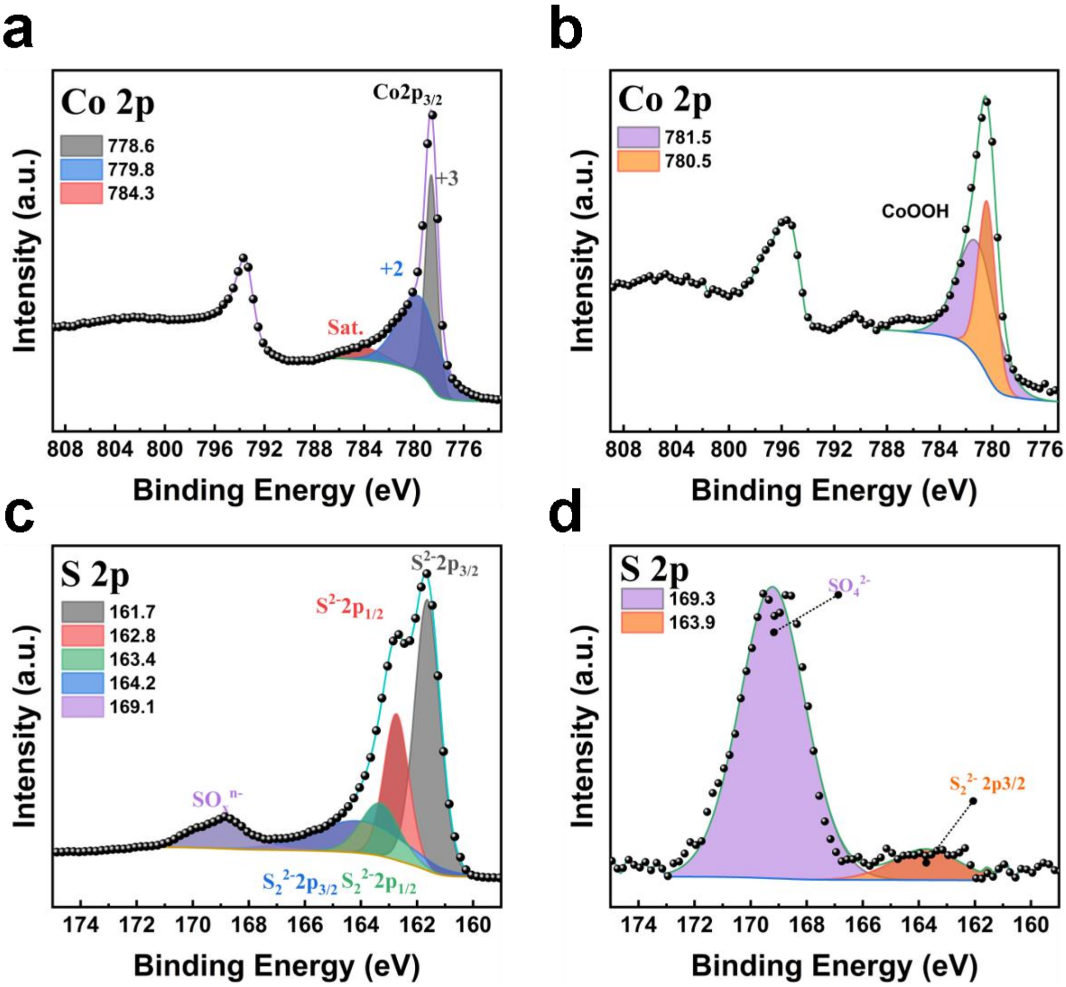

Figure S21. a-b) XPS spectra of Co 2p in H-Co_3S_4-B and H-Co_3S_4-A. c-d) XPS spectra of S 2p in H-Co_3S_4-B and H-Co_3S_4-A.

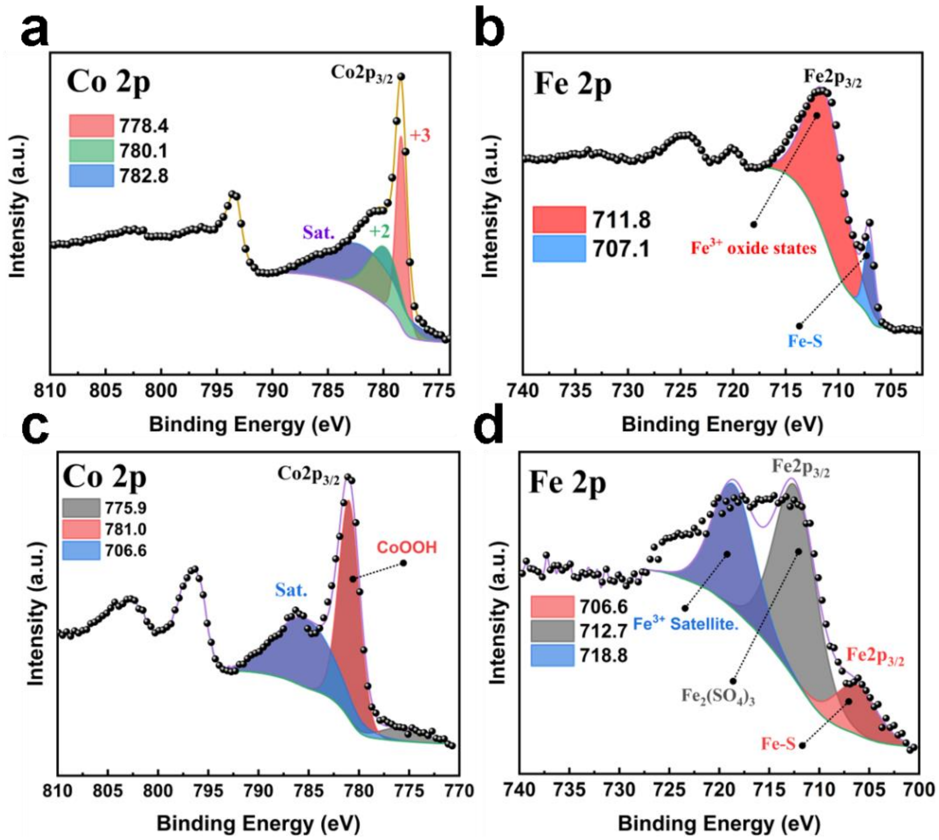

Figure S22. a-b) XPS spectra of Co 2p, and Fe 2p in H-$CoFeS_x$-B. C-d) XPS spectra of Co 2p, and Fe 2p in H-$CoFeS_x$-A.

Table S1. Simulation parameters.

Parameters	Value
Sphere diameter(D)	5×10^{-7} m
Bore diameter(d)	5×10^{-8} m
Hydroxide ions diffusion coefficient (D_{OH-})	5.273×10^{-9} m^2/s
Oxygen diffusion coefficient (D_{O2})	2.1×10^{-6} m^2/s
Cathodic charge transfer coefficient(α_c)	0.5
Anodic charge transfer coefficient(α_a)	0.5
Potential(φ_s)	1.3-1.65 V
Exchange current density(j_0)	0.1 mA/cm^2
Faraday constant(F)	96485 C/mol
Temperature(T)	298.15 K
Gas constant(R)	8.3145 J/(mol·K)
Hydroxide ions concentration in the bulk phase (C_{OH-}^0)	$100mol/m^3$
Oxygen concentration in the bulk phase (C_{O2}^0)	$2.648mol/m^3$

Table S2. EXAFS data fitting results of H-Co_3S_4-B, H-Co_3S_4-A and H-$CoFeS_x$-B. N, coordination number; R, distance between absorber and backscatter atoms; σ^2, the Debye-Waller factor value; ΔE_0(eV), inner potential correction to account for the difference in the inner potential between the sample and the reference compound.

Sample	Edge	Path	*N*	*R*(Å)	$\sigma^2(10^{-3}Å^2)$	ΔE_0(eV)	*R*-factor
H-Co_3S_4-B	Co	Co-S	4.1	2.23	6.5	-5.2	0.007
		Co-Co1	1.0	3.27	8.6	-5.2	
		Co-Co2	6.1	3.85	24.9	-5.2	
H-Co_3S_4-A	Co	Co-O	4.2	1.90	3.7	-3.1	0.008
		Co-Co	4.2	2.85	6.3	-3.1	
H-$CoFeS_x$-B	Co	Co-S	3.9	2.22	6.6	-7.5	0.015
		Co-Fe	0.3	2.52	4.8	-7.5	

Table S3. Summary of BET surface area, total pore volume and average pore diameter of the S-Co_3S_4, H-Co_3S_4, and H-$CoFeS_x$.

Sample	S_{BET} / m^2 g^{-1}	Pore volume / cm^3 g^{-1}	Average pore diameter / nm
S-Co_3S_4	1.28	0.0079	13.42
H-Co_3S_4	57.3	0.21	9.33
H-$CoFeS_x$	44.4	0.14	10.13

Table S4. The OER performance of H-CoFeS$_x$ as compared to the state-of-the-art catalysts in alkaline seawater. (FTO, fluorine-doped tin oxide; GCE, glass carbon electrode; NF, Nickle foam; CC, carbon cloth)

Samples	Electrolytes	Electrode	η@10 mA cm^{-2} (V)	η@100 mA cm^{-2} (V)	Tafel Slope (mV dec^{-1})	Mass Loading (mg cm^{-2})	Reference
H-CoFeS$_x$	1.0 M KOH + 0.6 M NaCl	GCE	1.437	1.465	29.8	0.026	This Work
Ni-doped FeOOH	1.0 M KOH + Seawater	NF	-	1.531	-	3.41	[3]
NiFe-LDH	1.0 M KOH + 0.5 M NaCl	NF	-	1.457	-	0.32	[4]
S-(Ni,Fe)OOH	1.0 M KOH + Seawater	NF	-	1.530	48.9	-	[5]
Ni_2P-Fe_2P	1.0 M KOH + Seawater	NF	-	1.811	-	15.0	[6]
Co-Fe-O-B	1.0 M KOH + 0.5 M NaCl	GCE	1.524	-	-	0.1	[7]
CoP$_x$@FeOOH	1.0 M KOH + Seawater	NF	1.465	1.513	50.3	4.1	[8]
$Pb_2Ru_2O_{7-x}$	0.1 M NaOH + 0.6 M NaCl	FTO	1.430	-	45	0.2	[9]
Co-Fe_2P	1.0 M KOH + 0.5 M NaCl	NF	-	1.69	-	2.0	[10]
RuV-CoNiP	1.0 M KOH + Seawater	NF	-	1.548	-	-	[11]
NiMoN@NiFeN	1.0 M KOH + 0.5 M NaCl	NF	-	1.516	-	1.27	[12]
$Na_2Co_{0.75}Fe_{0.25}P_2O_7$/C	0.1 M NaOH + 0.5 M NaCl	CC	1.515	1.600	53.0	-	[13]

References

[1] R. Y. Lai, A. J. Bard, *J. Phys. Chem. A* **2003**, *107*, 3335.

[2] D. A. Aikens, *J. Chem. Educ.* **1983**, *60*, A25.

[3] Y. S. Park, J. Lee, M. J. Jang, J. Yang, J. Jeong, J. Park, Y. Kim, M. H. Seo, Z. Chen, S. M. Choi, *J. Mater. Chem. A* **2021**, *9*, 9586.

[4] Q. Tu, W. Liu, M. Jiang, W. Wang, Q. Kang, P. Wang, W. Zhou, F. Zhou, *ACS Appl. Energy Mater.* **2021**, *4*, 4630.

[5] L. Yu, L. Wu, B. McElhenny, S. Song, D. Luo, F. Zhang, Y. Yu, S. Chen, Z. Ren, *Energy Environ. Sci.* **2020**, *13*, 3439.

[6] L. Wu, L. Yu, F. Zhang, B. McElhenny, D. Luo, A. Karim, S. Chen, Z. Ren, *Adv. Funct. Mater.* **2021**, *31*, 2006484.

[7] S. Gupta, M. Forster, A. Yadav, A. J. Cowan, N. Patel, M. Patel, *ACS Appl. Energy Mater.* **2020**, *3*, 7619.

[8] L. Wu, L. Yu, B. McElhenny, X. Xing, D. Luo, F. Zhang, J. Bao, S. Chen, Z. Ren, *Appl. Catal. , B* **2021**, *294*, 120256.

[9] P. Gayen, S. Saha, V. Ramani, *ACS Appl. Energy Mater.* **2020**, *3*, 3978.

[10] S. Wang, P. Yang, X. Sun, H. Xing, J. Hu, P. Chen, Z. Cui, W. Zhu, Z. Ma, *Appl. Catal. , B* **2021**, *297*, 120386.

[11] Q. Ma, H. Jin, F. Xia, H. Xu, J. Zhu, R. Qin, H. Bai, B. Shuai, W. Huang, D. Chen, Z. Li, J. Wu, J. Yu, S. Mu, *J. Mater. Chem. A* **2021**, *9*, 26852.

[12] L. Yu, Q. Zhu, S. Song, B. McElhenny, D. Wang, C. Wu, Z. Qin, J. Bao, Y. Yu, S. Chen, Z. Ren, *Nat. Commun.* **2019**, *10*, 5106.

[13] H. J. Song, H. Yoon, B. Ju, D.-Y. Lee, D.-W. Kim, *ACS Catal.* **2020**, *10*, 702.
